# Dynamics of Sentence Handwriting in Dyslexia: The Impact of Frequency and Consistency

**DOI:** 10.3389/fpsyg.2020.00319

**Published:** 2020-02-21

**Authors:** Paz Suárez-Coalla, Olivia Afonso, Cristina Martínez-García, Fernando Cuetos

**Affiliations:** ^1^Faculty of Psychology, University of Oviedo, Asturias, Spain; ^2^Department of Psychology, Health and Professional Development, Oxford Brookes University, Oxford, United Kingdom

**Keywords:** dyslexia, handwriting, sentence, orthographic consistency, lexical frequency, Spanish

## Abstract

Previous literature has indicated that linguistic and motor processes influence each other during written sentence production, and that the scope of this influence varies according to spelling ability or cognitive resources available. This study investigated how the spelling deficits associated with dyslexia affect the dynamics of the interaction between central and peripheral processes and the level of anticipation that can be observed in word spelling in the context of a sentence to dictation task. Children 9–12-year-olds with and without dyslexia wrote sentences to dictation in which the lexical frequency and phonology-to-orthography consistency of the last word (target) were manipulated. Analyses of kinematic measures (writing durations, in-air pen duration, and peaks of speed) revealed that children with dyslexia showed lexical frequency effects evident in within-word pauses (in-air pen) in the article and noun production. In addition, both children with and without dyslexia showed a phonology-to-orthography consistency effect in the pause before the target word. This effect tended to continue affecting the execution of the syllable prior to the inconsistency only in the group with dyslexia. Results support the influence of linguistic processes on motor execution. In addition, the study provides evidence of the impact of spelling deficits on the dynamics of handwriting in children with dyslexia.

## Introduction

Writing words involves both spelling processes (i.e., central processes) and graphomotor execution (i.e., peripheral processes). The nature of the spelling-motor interaction is still an unresolved issue. In fact, results from previous investigations are not consistent, in large part because varying tasks, orthographic systems, and/or measures have been employed (Søvik et al., 1994; Delattre et al., 2006; Lambert et al., 2011; Afonso et al., 2015a, b, 2018; Kandel and Perret, 2015a). However, accumulating evidence suggests that there is a complex relationship between both central and peripheral processes when writing and that this relationship may change with age, spelling and graphomotor skills (Olive and Kellogg, 2002; Sausset et al., 2012; Afonso et al., 2015a; Kandel and Perret, 2015b). It is well-documented that children and adults with dyslexia have persistent difficulties with accurate spelling (Wimmer and Mayringer, 2002; Lyon et al., 2003; Tops et al., 2012; Suárez-Coalla et al., 2016) that constrain handwriting production (Sumner et al., 2013, 2014; Afonso et al., 2015b). However, how the interaction between linguistic and motor processes is affected in children with dyslexia is still unknown, especially during the production of written words embedded in sentences, a very frequent task at school.

Although different mechanisms may be proposed to explain how linguistic processes affect motor processes (Afonso and Álvarez, 2019), a widely accepted explanation is that described by Olive (2014) in his model of cascade sentence writing. According to this account, different processes may be active in parallel during written production. Information processed at a certain level could flow (or “cascade”) from higher-levels of processing to affect lower-level processes, allowing higher-level modules to deal with forthcoming linguistic units while motor processes are engaged in the production of preceding segments. This would lead to concurrent activation of different writing processes, which would be engaged with different parts of a sentence. Thus, when words must be produced successively (like in a sentence), some characteristics or dimensions of words could be planned during the writing of previous words, while others would be handled after starting the motor execution of the word (Bonin et al., 2006; Lambert et al., 2011; Fayol and Lété, 2012; Maggio et al., 2012, 2015).

Support for this point of view has been obtained in several studies that have analyzed the kinematics of the written response. Lambert et al. (2011) explored how lexical frequency and phonology-to-orthography regularity affect the time-course of handwriting during a four-words copying task. Undergraduates had to write a sequence of four words, where the target word (of varying lexical frequency and regularity) was placed in third position. The authors tried to test whether the spelling of the target word takes place during graphomotor execution of the previous word, which would mean an effect of anticipation or, in contrast, whether the spelling occurs during the pause right before the target word. Results proved that adult writers are able to retrieve the spelling of a word during the graphomotor execution of the previous word, showing a clear anticipation effect. In the same vein, Maggio et al. (2015) performed a written denomination task, where French adults wrote words for which frequency, orthographic consistency, and length were manipulated and that were preceded or not by a determiner. Results indicated that the speed of noun production decreased in the condition without the determiner, as the intra noun pauses was longer in that condition. Moreover, retrieval of the noun’s spelling seemed to start before the determiner and continue during writing production. The frequency effect impacted on latencies and noun writing rate both with and without determiner; and the consistency effect was evident in the determiner writing rate, suggesting that some spelling features were being retrieved before or during the determiner production. Thus, the few works carried out so far with sentence writing about this issue indicate that there is a clear parallel processing and some anticipation effect when adults write successive words.

Crucially, according to Olive’s (2014) model if sufficient cognitive resources are not available, information may cease to flow leading to serial processing of the different units of the message (Alamargot et al., 2007, 2010; Lambert et al., 2011). Support for this notion has been found in different populations and using a range of tasks. Sausset et al. (2012) found that adults were able to process all the syllables of a word in parallel before they started producing a word if they were asked to write in lower-case print letters (i.e., a condition with low graphomotor constraints). However, syllabic processing became more serial (with each syllable being prepared at the corresponding inter-syllabic interval) if graphomotor constraints imposed by the task increased by asking the participants to write in a less familiar condition (e.g., large upper-case print without visual feedback).

Due to the high demands of both handwriting and spelling processes during early writing acquisition, it is reasonably to think that age may play an important role in the level of parallel processing and the time-course of anticipation during writing. This issue has been mostly analyzed in studies investigating the written production of isolated words (Bonin and Fayol, 2002; Delattre et al., 2006; Lambert et al., 2011; Sausset et al., 2012; Afonso et al., 2018; Suárez-Coalla et al., 2018). Evidence obtained has shown that the impact of some linguistic factors on handwriting dynamics is different for different age groups. Kandel and Perret (2015a) found that phonology-to-orthography consistency affected the writing durations produced by French children between 8 and 10 years of age, but that the effect was larger for the younger children. Regarding word frequency, it has been found that this variable affects written latencies in both children and adults (Delattre et al., 2006; Lambert et al., 2011; Afonso et al., 2018), but its influence on writing durations seems to depend on the age of the writer (Søvik et al., 1994; Kandel and Perret, 2015b; Afonso et al., 2018; Suárez-Coalla et al., 2018). Afonso et al. (2018) reported that the effect of lexical frequency in Spanish children strongly affected the writing durations of 8-year-olds, but this effect decreased to disappear by 11 years of age.

Research investigating how the effects of the influence of linguistic processes on handwriting movements are affected by developmental dyslexia is still rather limited. Nonetheless, the few studies addressing this issue have consistently found that spelling difficulties experienced by individuals with dyslexia modify the scope of the influence that lexical and sublexical processes have on writing durations. French children with dyslexia exhibited larger effects of phonology-to-orthography regularity and lexicality on writing durations than peers without dyslexia in a word copying task (Kandel et al., 2017). Interestingly, in a study conducted in Spanish, children with dyslexia showed larger effects of consistency and lexical frequency in written latencies than typical readers, but a reduced effect of word frequency on writing durations in spelling to-dictation than in copying (Afonso et al., 2019). This pattern of results may be reflecting a reduced ability to engage in parallel processing when spelling words to dictation in younger children compared to older children and in children with dyslexia than in their peers without dyslexia.

To sum up, research devoted to written production of isolated words (Delattre et al., 2006; Álvarez et al., 2009; Lambert et al., 2011; Sausset et al., 2012; Kandel et al., 2013, 2014; Roux et al., 2013; Buchwald and Falconer, 2014; Afonso et al., 2015b) supports the hypothesis that central and peripheral processes interact during writing and that this interaction varies as a function of age and spelling ability (Lambert et al., 2011; Sausset et al., 2012; Afonso et al., 2015b). Specifically, spelling difficulties experienced by individuals with dyslexia seem to affect the extent to which linguistic processes affect word writing (Kandel et al., 2017; Afonso et al., 2019). According to the assumptions made by Olive’s (2014) model, potential difficulties associated with the ability to parallel processing during writing should be more apparent when cognitive demands of the tasks are higher, like in a sentence production task or during text composition. The few studies that have investigated the effect of spelling difficulties associated with dyslexia on the production of linguistic units larger than a single word suggest that this may be the case (Berninger and Swanson, 1994; Sumner et al., 2013, 2014). In a study conducted with 9-year-olds, Sumner et al. (2013) observed that children with dyslexia produced a similar amount of letters per minute than their peers without dyslexia in an alphabet-writing task (a task with low cognitive demands), but they produced fewer words during text composition (a task with high cognitive demands). Children with dyslexia have also been reported to pause more often than peers in a sentence-copying task (Sumner et al., 2014), even if this task does not require generating ideas or retrieving the correct spelling of words. It seems that sentence-copying exerts sufficient cognitive demands to detect the effect of spelling difficulties on the handwriting movements produced by English-speaking children with dyslexia.

Spelling to dictation is a more cognitively demanding task than copying in at least two aspects. Firstly, during dictation writers must generate the spelling of the target words, while in copying the orthographic form is provided in the input. This may be a crucial point for individuals with spelling difficulties and may explain the reduced evidence for parallel processing observed in spelling to dictation when compared to copying in children with dyslexia (Afonso et al., 2019). Secondly, dictation requires maintaining the linguistic message in memory, while in a copying task this is usually available during the production of the response. This difference is more pronounced in sentence production than in single-word production tasks, since the linguistic message is substantially longer. Thus, differently from text composition tasks, writing sentences to dictation removes the demands related to idea generation but exerts specific demands on spelling processes. This makes this task especially interesting for the study of the dynamics of the relationship between spelling difficulties and handwriting performance. Surprisingly, no studies have approached the writing-to-dictation of words embedded in sentences.

Therefore, the main purpose of this study was to address the dynamics of handwriting in children with and without dyslexia, when they face a spelling-to-dictation of words embedded in sentences task. This task makes possible to explore the effects of spelling on handwriting, but eliminating the cognitive demands associated with planning or reading. The sentence structure was: article^1^ + noun^1^ + verb + preposition + article^2^ + noun^2^. The noun^2^ was the target word, where lexical frequency and orthographic consistency were manipulated. The last word in the sentence was chosen as the target position based on previous findings that demonstrate that in the spelling tasks writers start writing as soon as they identify the first sounds of the auditory input (Afonso et al., 2018, 2019). We aimed to make sure that participants would not be able to actually produce the word before the end of the stimulus or shortly after, which would reduce the possibility of observing effects related to increased cognitive load. As spelling difficulties seem to constrain the writing flow and the dictation task implies some cognitive load, it was expected that significant differences between children with and without dyslexia in the temporal characteristics of handwriting processing would be found. Specifically, larger frequency and consistency effects in children with dyslexia than in children without dyslexia were expected. In addition, and in line with previous literature, differences between the impact of lexical frequency and inconsistency on handwriting dynamics are expected, with consistency having a greater impact on writing durations. We predicted that if children with dyslexia will not be able to solve the inconsistency before they start handwriting, then spelling will overrun the pause between article^2^ and noun^2^ (target), and as a consequence, the graphomotor execution of the first syllable (before the inconsistency) will slow down.

## Materials and Methods

### Participants

A total of 36 children between 9 and 12 years of age (mean age 10 years; 8 months; *SD* = 0.9) participated in this study: eighteen with diagnosis of dyslexia (DYS) and 18 age-matched children without reading problems who served as controls (CON). Both groups consisted of 10 boys and 8 girls. They were also matched by socioeconomic status and type of handwriting (print letter or cursive writing). Participants with dyslexia were recruited from several primary schools, the Association of Dyslexia and certain Speech Therapy Centers of Asturias (Spain). They had previously received the diagnosis of dyslexia and they were receiving therapy in order to overcome or reduce their literacy difficulties. Participants without dyslexia were recruited from several primary schools in Asturias (Spain).

All of the participants were native Spanish speakers and had no known motor or perceptual disorders. They had an intelligence quotient (IQ) of 85 or higher according to the Wechsler Intelligence Scale for Children (Wechsler, 2001). Before performing the experimental tasks, a reading battery, PROLEC-R (Cuetos et al., 2014), was administered to all participants in order to assess reading performance and confirm reading difficulties in children with dyslexia. PROLEC-R yields scores (accuracy and total reading times) for word and pseudoword reading. The word section consists of 40 Spanish words, both high and low frequency. For each half, 10 words are short words and 10 are long words. The pseudoword section includes 40 pseudowords, half of them short and the other half long. Children with dyslexia (included in the DYS group) scored 1.5 to 2 standard deviations below the age norms provided by PROLEC-R in both accuracy and reading speed. Children without dyslexia (included in the CON group) had an age appropriate score in both sections. Means, standard deviations and *p* values for demographic characteristics and scores obtained in reading assessment tests are provided in Table 1.

**TABLE 1:** Means and standard deviations (in parenthesis) for demographic characteristics and reading scores of children with dyslexia (DYS) and chronological age-matched controls (CON).

	DYS *M* (*SD*)	CON *M* (*SD*)	*p*-value
Age (years)	10.8 (0.9)	10.7 (0.8)	*p* = 0.66
Education (years)	8.2 (0.8)	8.2 (0.8)	*p* = 1

**Reading**			

**Words**			
Accuracy (out of 40)	35.88 (2.72)	39.66 (0.59)	*p* < 0.001
Speed (s)	58.61 (21.25)	22.22 (3.35)	*p* < 0.001
**Pseudowords**			
Accuracy (out of 40)	32.00 (4.49)	38.61 (0.91)	*p* < 0.001
Speed (s)	78.50 (24.84)	41.55 (8.35)	*p* < 0.001

In addition, we collected data about spelling ability from children with dyslexia, using the spelling battery PROESC (Cuetos et al., 2002). This battery includes 25 inconsistent words, 25 ruled words and 25 pseudowords. The inconsistent words include at least two spelling options for one of its phonemes. The correct spelling of these words requires lexical knowledge and it is not enough to know the phoneme-grapheme conversion rules to spell them properly (e.g., “bolsa” [bag]). The ruled words includes some special orthographic rules (e.g., verbs ending in “-bir” must be written with “b” instead of “v,” as in “recibir” [receive]). Finally, the correct spelling of pseudowords must be derived from the phoneme-grapheme conversion rules, as we do not have an orthographic representation for them (e.g., “sirulo”). Children with dyslexia showed a very low performance in the spelling battery. Specifically, they scored a *M* = 14.55, *SD* = 3.95 for the inconsistent words (while the average for their age, according to battery norms, is between 21 and 23), *M* = 16.38, *SD* = 3.66 for the ruled words (the average for their age is between 23 and 24); and *M* = 17.72, *SD* = 2.19 for the pseudowords (the average for their age is between 24 and 25).

Regarding the number of participants, *post hoc* computations conducted with G^∗^Power 3.1.9.4 (Faul et al., 2009) of the achieved power showed that, given an α = 0.05 and a total sample size of 36, the power achieved in this study to detect a significant effect with an effect size η*^2^_*p*_* = 0.12 in a within-between interaction in a repeated measures ANOVA was 1 - β = 0.99. The effect size selected was that obtained in a significant interaction between the variable group (dyslexia versus typically-developing readers) and word frequency on the written latencies of adults of Spanish speakers (Afonso et al., 2015b).

### Materials

The experimental task consisted of a writing-sentence-to-dictation-task, where twenty-four sentences, six words each, were employed. The sentence structure was: article^1^ + noun^1^ + verb + preposition + article^2^ + noun^2^ (target word); and the sentences were classified in four conditions (six sentences in each condition), where the lexical frequency and orthographic consistency were manipulated for the noun^2^ of the sentences. On the other hand, the first syllable, the number of syllables, the number of letters number, the neighborhood size and the identity of the preposition and the article previous to the noun^2^ were controlled across the conditions. Consequently, considering the noun^2^ we had the following type of sentences: (1) high frequency and consistent words HFC - e.g., La gata descansa en el regazo [The cat rests in the lap], (2) low frequency and consistent words LFC - e.g., El marinero participa en la regata [The sailor participates in the regatta], (3) high frequency and inconsistent words HFI – e.g., La paisana pasea con el rebaño [The countrywoman walks with the flock], (4) low frequency and inconsistent words LFI – e.g., La tendera insiste en la rebaja [The shopkeeper insists on the rebate]. In addition, lexical frequency, orthographic consistency and length (syllable and letter number) were controlled for the noun^1^ and the verb.

For the lexical frequency manipulation, we used the values provided by ONESC (Martínez and García, 2008). This database (for orthographic neighbors and including lexical frequency) was created from the cumulative dictionary of the six grades offered in Martínez and García (2004), a dictionary of frequencies for written language in children 6–12 years of age. In the dictionary of Martínez and García (2004), authors tried to make a quasi-absolute database of the words, considering the words a group of children found in their reading. The number of words (and also the number of children) to create this database was small. However, we consider this database is suitable for this study. As the age range in the groups tested is considerable (9–12 years of age) it is very important for the present study to ensure that word frequency values used to select material are applicable to children attending a range of different grades. The lexical frequency for the HF words was *M* = 71.08 (*SD* = 78.05), and for the LF ones *M* = 5.15 (*SD* = 5.43).

The selection of the inconsistent words was based on the P-G rules of the first phoneme of the second syllable. In this case, all of the inconsistent words had, at the beginning of the second syllable, a phoneme with two alternative spellings. For example, the word no-**V**e-la ([no**’b**ela], novel) is inconsistent because the second syllable (-Ve-) starts with the phoneme/β/, which in Spanish could be spelled as V or B (e.g., novela -correct- vs. nobela -incorrect-). Words in which spelling decisions of the second syllable are context-dependent have been excluded (e.g., c, z). By contrary, the consistent words were selected when the first phoneme of the second syllable only included phonemes with unambiguous spellings, for example, no-**T**a-rio, [no’**t**ario], notary), where the phoneme/t/is represented by only one grapheme “t.” The full set of sentences with the values for manipulated and controlled variables is given in Supplementary Material Appendix A. For each sentence, an auditory stimulus was created for the spelling-to-dictation task.

### Procedure

Sentence presentation and digital recording of the responses were controlled by Ductus (Guinet and Kandel, 2010). The experiment was run on an HP Mini laptop. A WACOM Intuos 5 graphic tablet connected to the computer and an Intuos Inking Pen were used to register the participants’ responses. Auditory stimuli were recorded by a female speaker with a Plantronics microphone and edited with Audacity. The experimental sessions were carried out for each participant individually in a quiet room in the children school or private speech therapist center.

In the task, each trial started with the simultaneous presentation of an auditory signal and a 500-millisecond fixation point. The auditory stimulus was presented 500 milliseconds after offset of the fixation point. Participants had to listen to the auditory stimuli twice and then write the sentence in lower case, but with the first letter capitalized (as they typically do in the classroom), on a lined sheet of paper placed over the digitizer as quickly and as accurately as possible. When they finished the sentence, participants were asked to hold the pen over the next line of the response sheet, but without making any contact with the paper. In this moment, the experimenter clicked the left button of the mouse to start a new stimulus. The experimental session lasted around 20 minutes.

The research design was approved by the Ethics Committee for Research of the Principality of Asturias, Spain. The study was developed in accordance with the Declaration of Helsinki and the Spanish Law of Personal Data Protection (15/1999 and 3/2018) principles, and the data collection was covered by a written informed parental consent, obtained for all participants.

### Statistical Analysis

For the statistical analyses, in addition to accuracy, we considered several critical segments: the pause between the preposition and the article^2^ [e.g., La gata descansa EN ↔EL regazo], the article^2^ previous to the noun^2^ [e.g., La gata descansa en EL regazo], the pause between the article^2^ and the noun^2^ [e.g., La gata descansa en EL↔REGAZO] and the first syllable of the noun^2^ [e.g., La gata descansa en el REgazo]. The trajectory and tangential velocity were used to isolate the syllable, using geometric (cuspids and curvature maxima) and kinematic (velocity minima) criteria when necessary, as proposed by Kandel and Valdois (2006). In order to distinguish between increased cognitive load emerging during parallel processing and during serial processing, the total duration of a word was divided in on-paper writing duration (in which the pen is in contact with the paper) and in-air pen duration (the total time within a word that the pen did not make contact with the tablet). Thus, the considered measures were the writing durations (after excluding the in-air pen duration), the in-air pen durations (within the word or syllable and between-words pause), and the number of peaks of speed. As only correct responses for the noun^2^ were included in these analyses, responses with misspellings, self-corrections or missing data were removed from these analyses (in total, 19.33% of data were removed; 11.9% for the DYS group and 7.4% for the CON group). Besides, data above and below 2 standard deviations from the mean by participant and word were also excluded from the analysis (3.91%). For writing duration and in-air pen duration, ANOVAs were performed with mixed-effects analyses (Baayen, 2008) using R-software (RStudio, R Studio Team, 2015) with participants and items as random-effect variables and group, word frequency, and orthographic consistency as fixed factors. The most complex adjustment model (adjustment on the by-participants and by-item intercepts and by-participant slopes) was included in all the analyses (Barr et al., 2013). Stepwise model comparisons were conducted, from the most complex to the simplest model, and the one with the most complex adjustment but the smallest Bayesian information criterion (BIC) and significant χ^2^ test for the log-likelihood was retained (Schwarz, 1978). *F* values from the ANOVAs of type III, with Satterthwaite approximation for degrees of freedom, are reported for fixed-effects. If interactions were significant, *t*-tests were performed and the *p*-values were adjusted via the Holm-Bonferroni method. For the analyses of errors, we used a generalized mixed-effect model with a binomial distribution. A *p*-value < 0.05 was adopted as a level of significance.

## Results

### Writing Durations, In-Air Pen Durations, Peaks of Speed and Accuracy

Writing durations (in milliseconds) were considered as the time the pen was in contact with the tablet within a given word, so the in air-pen duration was excluded. In air-pen duration (in milliseconds) refers to the total time the pen is not in contact with the tablet for a given word. The number of peaks of speed or movement fluency involved the number of absolute velocity peaks in the velocity profile for each segment.

Writing durations, in-air pen durations, and number of peaks of speed were collected for the article^2^ and the first syllable of noun^2^. In addition, in-air pen durations were considered for the pause between the preposition and the article^2^ and between the article^2^ and the noun^2^.

In addition, the number of correct answers was analyzed.

### Pauses (In-Air Pen Duration)

#### Between the Preposition and the Article^2^

The orthographic consistency factor had a marginally significant effect in the analysis conducted on in-air pen duration (or pause) between the preposition and the article^2^, *F*(1,18.313) = 3.28, *p* = 0.08 (*Estimate* = 24.39, *SE* = 18.31). The time of the pencil in the air was longer when the noun^2^ contained an inconsistent grapheme than when it did not have it.

#### Between the Article^2^ and the Noun^2^

The main effect of orthographic consistency was significant in the analysis conducted on in-air pen duration (or pause) between the article and the noun^2^, *F*(1,21.13) = 4.39, *p* < 0.05 (*Estimate* = 43.080, *SE* = 18.82). Longer pauses were observed before the production of nouns including an inconsistent segment.

#### Article

The main effect of lexical frequency was significant in the analysis conducted on in-air pen duration (or pause) during the article handwriting, *F*(1,23.28) = 5.69; *p* < 0.05 (*Estimate* = 13.09, *SE* = 5.48); and the lexical frequency by group interaction was also significant *F*(1,591.03) = 3.70; *p* < 0.05. Pairwise comparisons showed that only the DYS group showed a significant effect of lexical frequency in-air pen durations, *t*(70) = 2.90, *p* < 0.05 (*Estimate* = 21.40, *SE* = 7.36). See Table 2.

**TABLE 2:** Writing duration and in-air pen duration for article and first syllable of children with dyslexia (DYS) and control group (CON).

	Article		First syllable			
		
	In-air pen		In-air pen		Writing duration	
			
	HF (*SE*)	LF (*SE*)	HF (*SE*)	LF (*SE*)	Consist (*SE*)	Incons (*SE*)
**CON**	30.43 (11.35)	39.51 (11.77)	45.54 (12.21)	44.36 (12.23)	549.93 (40.02)	548.08 (40.24)
**DYS**	35.21 (11.42)	60.91 (11.70)	43.11 (12.44)	58.55 (12.39)	593.34 (40.25)	650.74 (40.61)

*HF, high frequency; LF, low frequency; Consist, orthographic consistent words; Incons, orthographic inconsistent word; SE, standard error.*

#### First Syllable of the Noun^2^

In addition, lexical frequency significantly affected in-air pen duration (or pause) during the first syllable handwriting, *F*(1,571.45) = 3.80, *p* < 0.05, (*Estimate* = 7.13, *SE* = 3.65); the interaction between lexical frequency and group was also significant, *F*(1,571.45) = 5.16; *p* < 0.05. Pairwise comparisons showed that only the DYS group showed a significant effect of lexical frequency, *t*(572) = 2.81, *p* < 0.05, (*Estimate* = 15.43, *SE* = 5.49). See Table 2.

### Writing Durations

#### Article^2^

The main effect of group was significant in the analysis conducted on writing durations, *F*(1,33.8) = 4.257, *p* < 0.05 (*Estimate* = 82.97, *SE* = 40.21). Children in the DYS group produced longer writing durations than children in the CON group.

#### First Syllable of the Noun^2^

A significant interaction between orthographic consistency and group was found on the writing durations analysis, *F*(1,547.33) = 11.69; *p* < 0.001. Pairwise comparison showed that orthographic consistency did not affect the CON group and affected marginally the DYS group *t*(31) = 2.55, *p* = 0.07 (*Estimate* = 57.39, *SE* = 22.47).

### Number of Peaks of Speed

The variable group only affected the number of peaks of speed in the article^2^ *F*(1,33.81) = 4.26, *p* < 0.05, (Estimate = 0.27, *SE* = 0.28), the DYS group showed more peaks of speed than the CON group.

### Target Noun Spelling Accuracy

Table 3 shows the mean percentage of correct responses and standard deviations in each condition for both groups. Considering the 19.33% of misspellings, self-corrections and missing data, we found 15.29% of misspellings (9.32% for the DYS group, and 5.97% for the CON group), 2.19% of self-corrections (1.73% for the DYS group, and 0.46% for the CON group), and 1.85% of missing data (only for the DYS group). We found different type of mistakes: (1) grapheme substitution that implies a phonologically plausible mistake [e.g., “ca**b**erna” instead of “ca**v**erna”]; (2) grapheme substitution that implies a phonologically non-plausible mistake, resulting in a pseudoword [e.g., “necano” instead of “decano”]; (3) semantic substitution [e.g., “lavabo” instead of “lavadero”]; (4) grapheme omission [e.g., “decan” instead of “decano”]; (5) mixed mistakes [e.g., “**r**e**b**oto” instead of “**d**e**v**oto”].

**TABLE 3:** Mean percentage of correct responses and standard deviations (in parenthesis) in each condition for both groups.

	% HF Consist	% LF Consist	% HF Incons	% LF Incons
**CON**	96.29 (9.14)	90.74 (8.52)	82.40 (16.64)	71.29 (12.53)
**DYS**	96.07 (7.29)	90.74 (11.74)	62.96 (20.25)	54.63 (18.79)

*HF Consist, high frequency and orthographic consistent words; LF Consist, low frequency and orthographic consistent words; HF Incons, high frequency and orthographic consistent words; LF Incons, low frequency and orthographic consistent words.*

The analysis showed an orthographic consistency effect (*p* < 0.01; Estimate = 1.56, *SE* = 0.57; *OR* = 0.21), and orthographic consistency by group interaction (*p* < 0.05, Estimate = 1.09, *SE* = 0.45; *OR* = 0.33), *post hoc* analysis revealed that the differences between groups was only significant for the inconsistent words (*p* < 0.001, Estimate = 1.08, *SE* = 0.26); in addition the consistency effect was larger for the DYS group (*p* < 0.001, Estimate = 2.64, *SE* = 0.57) than for the CON group (*p* < 0.05, Estimate = 1.55, *SE* = 0.58).

### Summary of the Results

In comparison to typically developing peers, children with dyslexia produced longer writing durations and more peaks of speed in the article preceding the target word. They also showed a larger effect of word frequency in the in-air pen durations produced within the article and the first syllable of the target noun. Moreover, the duration of the pause previous to the target word was similarly affected by orthographic consistency in both the group with dyslexia and the group without dyslexia. However, this effect lasted for longer in the group with dyslexia, also affecting writing durations during the production of the target word.

## Discussion

The goal of this study was to better characterize the dynamics of handwriting processes of children with DYS when they spell to dictation words embedded in a sentence. To achieve this objective, the time-course of sentence handwriting was considered. In this task, Spanish children with DYS wrote on a digitizer 24 sentences with the same structure, but in which the orthographic consistency and lexical frequency of the last word was manipulated. We compared their performance with that of chronological-age matched children without literacy problems. Writing durations, in-air pen durations, peaks of speed, and accuracy were analyzed.

Results revealed interesting information about handwritten words in the context of a dictated sentence. Differences between groups were evident, supporting the idea that spelling difficulties impact on accuracy and handwriting execution. Children with DYS made more errors than the CON group in the inconsistent words and they showed a larger lexical frequency effect than CON children on in-air pen duration during the article and first syllable production. The orthographic consistency effect seems to marginally continue impacting the production of the first syllable in DYS children. The effects of both frequency and consistency variables had different time courses during handwriting production.

The lexical frequency effect appeared in the article^2^ (before the target word) for children with DYS and continued along the first syllable of the noun^2^. Specifically, DYS children spent more time with the pen in the air (for both the article^2^ and the first syllable of the noun^2^) when they had to deal with LF words than when HF words were concerned. On the contrary, the CON group did not show a lexical frequency effect. In this study, where words were embedded in a sentence, DYS children showed an anticipation effect, as the frequency of a word had an effect on the production of the previous article^2^. These results confirm the difficulty for the DYS group accessing or processing low frequency words (Rüsseler et al., 2003; Afonso et al., 2015b, 2019), considering the small reading exposure they have. The extent to which this effect may be related to reduced exposure to written language in the group with dyslexia and thus, to differences between groups in the frequency with which words are actually encountered, is an issue that requires further investigation.

Similar to the effect found here in the previous article, Lambert et al. (2011) also reported anticipation of the word frequency effect in adult writers, as the spelling of one word seemed to be processed during the handwriting of the previous word. They considered that the graphomotor execution in adults implies a low cognitive load, and because of that, writers are able to process the spelling of the following word. In our case, we only found this anticipation effect for DYS children when they deal with LF words. Discrepancies could be due to several factors. First, they may be due to differences between the tasks used. While in our study we used a sentence-to-dictation task (with 6 words) and where the sentence was pronounced twice, the task used by Lambert et al. (2011) consisted of a four-words copying task, with the target word in third position. In this sense, it may be that the semantic context provided by the sentence in our study had contributed to a facilitation of the lexical selection process (Bonin et al., 2015) in the control group, leading to little difference between high-frequency and low-frequency words.

In previous research, the impact of lexical frequency on hand movements (e.g., writing durations) have yielded mixed results. In general, studies conducted with children have reported some effects of lexical frequency on writing durations (Søvik et al., 1994; Kandel and Perret, 2015a; Afonso et al., 2019), but not those carried out with adults (Delattre et al., 2006; Lambert et al., 2011). According to this, it seems that the influence of lexical frequency on writing durations may depend on the age or the spelling ability of the writer (Kandel and Perret, 2015a; Afonso et al., 2018, 2019). Previously reported results support the idea that lexical frequency modulates motor execution during writing acquisition (Afonso et al., 2018) and in children with dyslexia (Kandel et al., 2017), which is in line with our results. Interestingly, the frequency effect observed for the group with dyslexia affected the in-air pen durations produced within-words (namely, within the article and the first syllable of the target noun) rather than on-paper writing durations. This pattern may reflect a reduced ability to process in parallel the spelling of the word and the concurrent handwriting movements in the group with dyslexia. For children with dyslexia, information may cease to flow due to the cognitive demands exerted by the spelling to dictation task, leading to serial processing of central and peripheral processes (Olive, 2014). Accordingly, lexical access could only take place in pauses between periods of execution of writing movements.

Although this explanation seems to fit previous evidence (Alamargot et al., 2007, 2010; Lambert et al., 2011; Kandel et al., 2017) and widely accepted theoretical proposals developed in the field of writing (Olive, 2014), it is important to note that a strictly serial model could also accommodate the findings reported here. It has been suggested that individuals with dyslexia may experience difficulties accessing phonological or visual representations only when particularly high demands are imposed on short-term memory or when the task is especially challenging (Ramus and Szenkovits, 2008). In these cases, a processing bottleneck may occur for difficult stimuli, such as low-frequency words or inconsistent words. This bottleneck would lead to a postponement of the central processing for forthcoming units (Ferreira and Pashler, 2002), with the effects produced by the processing of previous units lasting more for students with dyslexia. This may also explain the word frequency effect in the present study. In any case, our findings clearly establish that the dynamics of the interaction between central and peripheral processes are altered in the handwriting production of Spanish children with dyslexia when compared to that of typically readers of the same age.

Alternatively, Spanish is a language with a more transparent orthography than French, so it is not impossible that our participants had relied more on the application of phonology-to-orthography conversion procedures than on lexical processes. This latter explanation may find support on the fact that our control group did show significant effects of orthographic consistency. Finally, it is also possible that the differences between high-frequency and low-frequency words are small in this group and that resources are enough to process low-frequency words without producing a significant impact on writing durations. In any case, more research is necessary in order to know more about the variables that affect the impact of word frequency in different tasks and populations.

Contrary to lexical frequency effect, orthographic consistency affected the pause between the preposition and the article^2^ (marginally), and the pause between the article^2^ and the noun^2^ in both DYS and CON children. However, this effect tended to last longer in the DYS group, affecting also (marginally) the writing durations of the first syllable of the noun^2^ in DYS children. As reported several times in studies using single words, orthographic consistency and regularity increases written latencies in adult and children (Bonin et al., 2001; Kandel and Valdois, 2005; Delattre et al., 2006; Roux et al., 2013; Afonso et al., 2015a, b; Suárez-Coalla et al., 2018). Crucially, the consistency and regularity effects seem to spread to affect the production of hand movements, indicating that inconsistencies are not fully resolved before writing starts (Roux et al., 2013; Afonso et al., 2015a, 2018, 2019; Kandel et al., 2017; Suárez-Coalla et al., 2018).

Moreover, this effect has been reported to depend on the position of the inconsistency (Roux et al., 2013; Suárez-Coalla et al., 2018) and on spelling ability (Afonso et al., 2015b). Recently, Kandel et al. (2017) found that irregularity increased writing duration and dysfluency in both children with and without dyslexia (ages 10–11), but the impact of regularity was larger for the group with dyslexia. In relation to our data, one may consider that CON children solve the inconsistency before they start the motor execution, during the previous pause.

However, the spelling processes were marginally active when DYS children were writing. According to this, and in accordance with accuracy results, difficulties with inconsistent words were more evident in DYS children than in CON ones. The absence of the effect of consistency in children without dyslexia may seem striking, but perhaps the semantic context provided by the sentence and the repeated presentation of the stimuli may have facilitated the resolution of the inconsistency before writing starts, thus favoring the disappearance of this effect on writing durations in this group of children.

Taken together the effects of orthographic consistency and lexical frequency, we observed that these variables produce different movement patterns in DYS children. The lexical frequency implied larger effects in in-air pen duration, while orthographic consistency impacted movement production. Similar results were reported by Kandel et al. (2017), suggesting that orthographic irregularities could have a stronger link with handwriting movements than lexical frequency. Further research addressing why these variables seem to have a different relationship with peripheral processes would surely provide valuable information to better understand the time-course of the different spelling routes.

In conclusion, our study provides evidence of the impact of linguistics variables on the peripheral processes during a sentence handwriting task, where DYS and CON children received words embedded in a sentence by dictation. Namely, we observed that the spelling deficit had an impact on the dynamics of sentence handwriting in dyslexia, as some differences between groups were found. Specifically, DYS children showed a word frequency effect evident in the article and the noun production. This frequency effect was manifested in within-word pauses (in-air pen), which is consistent with the idea of parallel processing of lexical and peripheral processes in individuals with dyslexia. In addition, both children with and without dyslexia showed a phonology-to-orthography consistency effect in the pause before the target word, but this effect continued to marginally affect the execution of the syllable prior to the inconsistency only in the group with dyslexia. This pattern supports the hypothesis that spelling impairment causes differences between children with dyslexia and age-matched peers in the dynamics of their writing, even when the planning and reading demands of the task are eliminated or reduced to a minimum.

Definitely, this study offers the opportunity to think over the spelling-motor interaction in children with and without spelling difficulties, as we tried to reach the impact of linguistic variables on graphomotor execution. Moreover, an effort was made to understand this interaction in the context of a sentence to dictation task, a very common classroom activity, but not very often used in research. This task seems to be suitable to achieve the effect of spelling difficulties on the handwriting movements, where information is not available during the response production (copying task) and generation of ideas is not necessary (text production). From our results, apart from the possible interpretations, it is clear that there are differences between Spanish children with and without dyslexia, in the dynamics of the spelling-motor interaction in the handwriting production.

The findings reported here have several implications for teachers of children with dyslexia. Accordingly, DYS children will need more time to successfully perform any written task including low frequency and inconsistent words. In this sense, adaptations may need to be considered at schools in order to facilitate the work of these children and avoid frustration. In addition, it should be important to help them to achieve writing accuracy.

## Data Availability Statement

The datasets generated for this study are available on request to the corresponding author.

## Ethics Statement

The studies involving human participants were reviewed and approved by the Ethics Committee for Research of the Principality of Asturias, Spain. Written informed consent to participate in this study was provided by the participants’ legal guardian/next of kin.

## Author Contributions

PS-C and OA developed the study concept and design. CM-G performed testing and data collection. PS-C and OA analyzed the data. PS-C, OA, and FC drafted the manuscript.

## Conflict of Interest

The authors declare that the research was conducted in the absence of any commercial or financial relationships that could be construed as a potential conflict of interest.
