# Supplementary material for: Dynamics of Sentence Handwriting in Dyslexia: The Impact of Frequency and Consistency
Source: Front Psychol. 2020 Feb 21;11:319. doi: 10.3389/fpsyg.2020.00319 (PMC7047968; doi:10.3389/fpsyg.2020.00319)
Supplement: Supplementary file 1 [file Data_Sheet_1.PDF]

Appendix A.

Stimuli characteristics and sentences.

		HF	Nsyll	Nlet	N-size		LF	Nsyll	Nlet	N-size
**Inconsistent**	**ca**verna [*cavern*]	28.1	3	7	0	**ca**becero [*headboard*]	0.99	4	8	1
	**na**vaja [*pocket knife*]	32.91	3	6	1	**na**biza [*cabbage type*]	1.96	3	6	0
	**no**vela [*novel*]	49.8	3	6	1	**no**vata [*rookie*]	6.54	3	6	2
	**re**baño [*flock*]	49.61	3	6	3	**re**baja [*sale*]	2.62	3	6	2
	**de**bate[*discussion*]	22.49	3	6	3	**de**voto [*devotee*]	1.11	3	6	1
	**la**berinto [*maze*]	41.2	4	9	0	**la**vadero [*laundry*]	6.87	4	8	0
**Consistent**	**ca**nario [*canary*]	47.1	3	7	1	**ca**nica [*marble*]	12.77	3	6	5
	**na**ranja [*orange*]	105	3	7	1	**na**dadora [*swimmer*]	0.85	4	8	0
	**no**ticia [*news*]	307.27	3	7	1	**no**tario [*notary*]	17.81	3	7	2
	**re**gazo [*lap*]	45.19	3	6	4	**re**gata [*regatta*]	1.34	3	6	6
	**de**porte [*sport*]	86	3	7	0	**de**cano [*dean*]	1.36	3	6	3
	**la**guna [*lagoon*]	48.67	3	6	0	**la**picero [*pencil*]	7.44	4	8	1

HF: high lexical frequency. LF: low lexical frequency. Nsyll: number of syllables. Nlet: number of letters. N-size: number of orthographic neighbours.

Sentences

El toro duerme en la caverna [*The bull sleeps in the* cave].
El panadero corta con la navaja [*The baker cuts with the pocket knife*].
La niña disfruta con la novela [*The girl enjoys the novel*].
La paisana pasea con el rebaño [*The countrywoman walks with the flock*].
El amigo pierde en el debate [*The friend loses in the debate*].
La tortuga entra en el laberinto [*The turtle enters the maze*].
El dueño pinta en el cabecero [*The owner paints in the headboard*].
El padre tropieza con la nabiza [*The father stumbles on the cabbage*].
La camarera pelea con la novata [*The waitress fights with the rookie*].
La tendera insiste en la rebaja [*The shopkeeper insists on the sale*].
El cura reza con el devoto [*The priest prays with the devotee*].
La señora reposa en el lavadero [*The lady rests in the laundry*].
El señor sale con el canario [*The lord goes out with the canary*].
El mono sueña con la naranja [*The monkey dreams of the orange*].
La maestra goza con la noticia [*The teacher enjoys the news*].
La gata descansa en el regazo [*The cat rests on the lap*].
El chico cree en el deporte [*The boy believes in sport*].
La pelota flota en la laguna [*The ball floats in the lagoon*].
El perro juega con la canica [*The dog plays with the marble*].
El anciano merienda con la nadadora [*The old man snack with the swimmer*].
La madre confía en el notario [*The mother trusts the notary*].
El marinero participa en la regata [*The sailor participates in the regatta*].
El estudiante discute con el decano [*The student discusses with the dean*].
La chica piensa en el lapicero [*The girl thinks about the pen*].
